# Extensive diversity of unusual microorganisms associated with severe pneumonia in kidney transplant recipients

**DOI:** 10.1371/journal.ppat.1013667

**Published:** 2025-11-03

**Authors:** Jia-Xin Lv, Yuan-Yuan Pei, Cheng Yang, Xiang Liu, Min-Jie Ju, Edward C. Holmes, Yan-Mei Chen, Tong-Yu Zhu, Yong-Zhen Zhang

**Affiliations:** 1 State Key Laboratory of Genetics and Development of Complex Phenotypes, School of Life Sciences, Human Phenome Institute, School of Public Health, Greater Bay Area Institute of Precision Medicine (Guangzhou), Fudan University, Shanghai, China; 2 Department of Kidney Transplantation, Shanghai Key Laboratory of Organ Transplantation, Zhongshan Hospital, Fudan University, Shanghai, China; 3 Zhangjiang Institute of Fudan University, Shanghai, China; 4 Department of Critical Care Medicine, Zhongshan Hospital, Fudan University, Shanghai, China; 5 School of Medical Sciences, The University of Sydney, Sydney, Australia; Wuhan Institute of Virology, Chinese Academy of Sciences(CAS), CHINA

## Abstract

Although pneumonia is a common lung disease with a high morbidity and mortality, aside from well-known pathogens little is known about why, which and how many microorganisms are associated with the disease, particularly in immunocompromised individuals. We enrolled 32 kidney transplant cases with severe pneumonia admitted to Shanghai Zhongshan Hospital between 2019 and 2025, and performed both metagenomic and metatranscriptomic sequencing on the bronchoalveolar lavage fluid (BALF) and blood samples from each case. Comprehensive analyses of immune cells and cytokines, as well as BALF and blood metatranscriptomes, revealed that both adaptive and innate immunity inside and outside of their lungs were severely suppressed. Notably, a high diversity of unusual microorganisms were present in BALF samples, including bacteria and DNA viruses that are rare or absent in healthy individuals, as well as RNA viruses and fungi. Of these, 17 bacteria, 46 DNA viruses, eight RNA viruses and two fungi, which were at high abundance, were considered to be responsible for the lung infections. Remarkably, the majority of these patients experienced co-infections of multiple bacteria, DNA and RNA viruses and fungi, reaching 32 virus species in one individual. In sum, these data indicate that the prosperity or overgrowth of accidental, opportunistic and rare microorganisms within the lungs of these kidney transplant patients substantially altered their lung microbiota, with multiple co-infections further exacerbating the severity of pneumonia.

## Author summary

Pneumonia is a primary contributor to morbidity and mortality in all age of groups globally, especially immunocompromised individuals. However, the definitive diagnosis of pneumonia still presents a major challenge to date due to its complexity and heterogeneity. In particular, little is known about the change in the lung microbiome in kidney transplant recipients (KTRs) with pneumonia. Our study revealed that both adaptive and innate immunity inside and outside of the lungs of KTRs with pneumonia were severely suppressed. In addition, their lung microbiomes varied greatly and were enriched with extensive diversity of unusual microorganisms including bacteria, fungi, DNA and RNA viruses. Co-infection of these unusual microorganisms was responsible for severe pneumonia in the majority of the KTRs. Our findings indicate that the severely impaired immunity in KTRs leads to significant alterations in the lung microbiome and co-infections with diverse unusual microorganisms, resulting in severe pneumonia and adverse outcomes. This provides new insights into lung infections, the microbiome, and immunity in KTRs.

## Introduction

Pneumonia is a common lung disease with high morbidity and mortality, particularly in younger children and older adults due to their relatively weakened immune defenses [1,2]. The profound impact of pneumonia was underscored by the COVID-19 pandemic [3]. Pneumonia is also a complex and heterogenous disease associated with a broad range of microorganisms and host responses [1,4]. Hence, the definitive diagnosis of pneumonia has presented a major challenge, leading to many undiagnosed cases [5,6] that in turn impact both treatment and clinical outcome. Furthermore, little is known about which and how many microorganisms co-occur in pneumonia in addition to well-known pulmonary pathogens, particularly in immunocompromised hosts. Despite advances in diagnostics, treatment, and prevention in recent decades, the mechanisms underlying pneumonia are also understudied [4,6].

Healthy lungs harbor a high diversity of microorganisms that experience transiently dynamic fluxes of immigration and clearance [7–10]. Clinical and experimental investigations have demonstrated the importance of a dynamic and balanced lung microbiome in the maturation and maintenance of homeostasis in respiratory physiology and immunity, as well as in the prevention of diseases [8–12]. In contrast, disruptions from the balanced microbiome (i.e., dysbiosis) are believed to be associated with pneumonia and other lung diseases, and characterized by different taxonomic signatures of microorganisms [13–17]. To date, however, there has been no consensus on whether the alteration of lung microbiota initiates or perpetuates inflammation and/or injury, or is simply a consequence and marker of pneumonia [9,18]. In addition, despite some understanding of the interplay between the microbiome and the host immune system, it is unclear how the immune system impacts lung microbial homeostasis, particularly in immunocompromised population. Finally, compared to bacteria, the viral and fungal communities present in lungs are relatively understudied [18].

Although kidney transplantation greatly improves the survival and quality of life of kidney transplant recipients (KTRs), like other solid organ transplant (SOT) recipients, they are more prone to infections from a wide range of microorganisms, including opportunistic pathogens [19–21]. Among post-transplant infections in KTRs, pneumonia emerges more frequently and is also a leading cause of the mortality [21–23], likely accounting for 45% of total infection-related KTR deaths. Like other SOT recipients, KTRs usually do not exhibit the typical clinical features apparent in immunocompetent individuals [20,24], which in turn impacts clinical diagnosis and anti-microbial treatments. In addition, although the number of global kidney transplants is now 102,117 annually, accounting for >65% of total SOT [25], little is currently known about the lung microbiome of KTRs. Indeed, we do not fully understand the signature of the lung microbiome and the mechanisms responsible for rendering KTRs more susceptible to pneumonia by various microorganisms, including those that are rare or absent in immunocompetent individuals. Finally, due to their immunocompromised state, investigating the lung microbiome and infectome of KTRs may offer a window to clarify the association between lung infection and microbiome, as well as providing important data on the impact of host immunity on lung microbial homeostasis.

To better understand pneumonia in KTRs, we enrolled 32 KTR cases presenting with severe pulmonary infection that were admitted to the Shanghai Zhongshan Hospital between 2019 and 2025. By characterizing their lung microorganisms and immune status inside and outside of lungs we determined all pathogens likely responsible for pneumonia. We also explored the mechanisms underlying disease in these immunocompromised patients.

## Results

### Patient cohort

**Fig 1:**
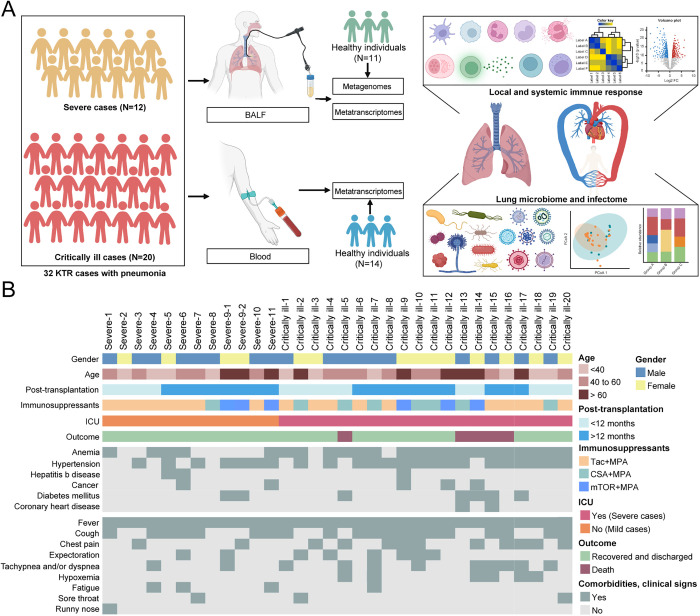
Study design and patient cohort. (A) Schematic summary of the study design and patient cohort. A total of 32 KTRs were enrolled. Bronchoalveolar lavage fluid (BALF) and whole blood samples were collected from each patient for detailed analysis of the immune responses and microbiome (including virome). Some illustrations were created using BioRender (https://biorender.com/). (B) Demographic and clinical information of the patients. Intensive care unit, ICU; tacrolimus, Tac; cyclosporin A, CsA; leflunomide, Lef; mycophenolic acid, MPA; mammalian target of rapamycin, mTOR.

We enrolled 31 KTR patients (32 cases) who were clinically diagnosed with severe pneumonia by chest computed tomography (CT) scan and hospitalized at the Zhongshan Hospital in Shanghai, China during 2019–2025 (Fig 1A and S1-S5 Tables). Notably, one patient suffered a second bout of pneumonia 10 months after the first infection, which we subsequently considered as two cases (denoted Severe-9–1 and Severe-9–2). These patients included 18 males and 13 females, with ages ranging from 23 to 71 years. All patients received immunosuppressants, with the treatment durations ranging from nine days to 396 months (Fig 1B). Of note, 19 patients ceased to take immunosuppressants during anti-infection, while five patients received a reduced dose. A total of 29 of the 31 patients (93.55%) suffered from comorbidities, with the most common diseases being anemia (n = 23) and hypertension (n = 22).

Of these cases, pneumonia occurred within 6 months post-transplant in five cases, between 6–12 months in nine cases, and >12 months in 18 cases, including one 33 years post-transplant. The intervals from infection to hospital admission ranged from 0-35 days. A total of 28 cases acquired the infection within their communities, with the remainder contracting the infection during hospitalization. Finally, all 32 cases received antibiotics, including meropenem and moxifloxacin, before admission or sample collection.

### Clinical features and outcome of KTRs with pneumonia

Despite the large variation in the intervals from infection onset to admission, fever (temperature >37.5℃) still appeared in all these cases at admission. Other signs included cough (60.00%), expectoration (26.67%), chest stuffiness and/or pain (36.67%), tachypnea and/or dyspnea (33.33%), hypoxemia (20.00%), and fatigue (13.33%) (S1 Table and Fig 1B). Chest CT scanning revealed abnormal images in all cases, with bilateral involvement in 26 cases and unilateral involvement in four cases (S1 Fig). Despite the discrepancies in the descriptions by three clinicians (see Methods), their imaging manifestations were mainly characterized with patchy shadows, nodular shadow, ground glass opacity, pleural effusion, and consolidation. The images still provided clues for differential diagnoses even after the determination of the infectious agents involved. For example, nearly all *Pneumocystis jirovecii* pneumonia cases exhibited bilateral interstitial pneumonia with diffuse ground-glass opacities, and became more complex, exhibiting additional patchy or diffuse areas of consolidation when accompanied by concomitant bacterial and/or viral agents. For patients without *P. jirovecii* infection, local patchy shadowing or consolidation was the most common CT finding.

Although all these KTR patients exhibited severe pneumonia, the severity of the illness in these KTRs varied substantially. Twenty cases appeared critically ill and were transferred into intensive care unit (ICU), while the remaining 12 cases remained in regular wards. Based on their severity and the need for intensive care, they were referred to as the ‘critically ill’ and ‘severe’ groups, respectively. Fortunately, 26 patients recovered or improved greatly and were discharged following treatment. However, the remaining five patients (all critically ill) died, comprising four who died in ICU despite rescue efforts and one who died at home after his relative abandoned treatment and requested discharge. The details of their clinical records were provided in S1-S5 Tables.

### Characterization of immune status of the KTRs

#### Systemic immunity in KTRs.

Although lung infections were present in all the KTRs, white cell counts were only elevated in nine cases and remained within the normal range in 20 cases (even decreasing in three cases) (S5 Table). Specifically, neutrophils and monocytes had irregular changes, eosinophils and lymphocytes were below normal range in the majority of these cases, while basophils remained within normal range in all cases. The percentage of lymphocyte subsets also exhibited irregular changes. For cytokines, the level of tumor necrosis factor alpha (TNF-α) was elevated in 26 cases and remained normal in five cases. Higher levels of both IL-6 and IL-2R appeared in 20 and 21 cases, respectively. However, the levels of IL-1β, IL-8 and IL-10 remained normal in most of these cases and only became higher in a few cases.

**Fig 2:**
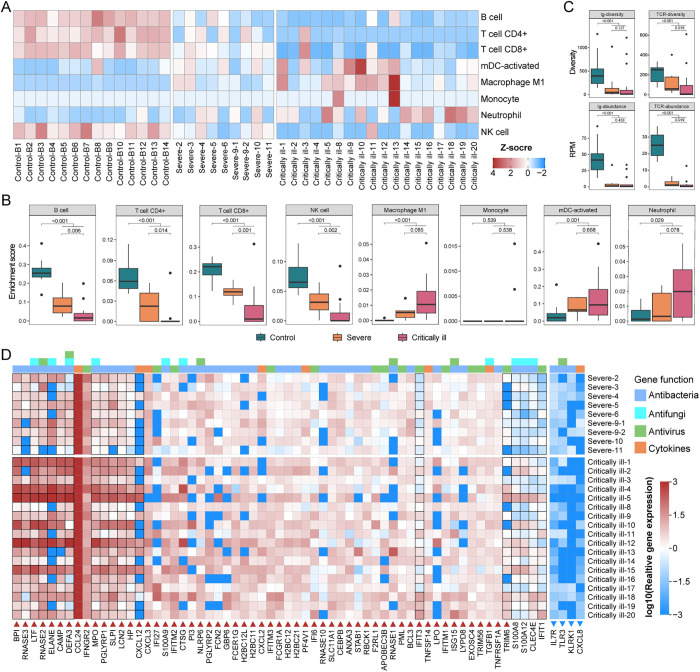
Systemic immune response in KTR cases with pneumonia. Cell type enrichment analysis of the whole-blood metatranscriptomes from KTRs and healthy individuals using X‐Cell. The colors of the heatmap represent the Z‐scaled values (deviation from the mean across all samples), with warmer (red) colors indicating positive values (enrichment) and cooler (blue) colors indicating negative values (depletion) for each cell type. (B) Boxplots indicating the enrichment score of eight immune cell calculated by X‐Cell in whole-blood metatranscriptomes from healthy individuals, severe cases and critically ill cases. Differences between groups were estimated using a Mann-Whitney U-test. The horizontal box lines in the boxplots represent the first quartile, the median, and the third quartile. The whiskers cover all data points within 1.5 times the inter-quartile range and the black points denote outliers. Myeloid dendritic cells, mDCs. (C) Boxplots indicating the abundance and diversity of Ig- and TCR-related genes in whole-blood metatranscriptomes from healthy individuals, severe cases and critically ill cases. (D) Heatmap indicating the relative expression of 65 significant differentially-expressed genes related to antimicrobial response pathways and the cytokine in whole-blood metatranscriptomes from KTR cases and control individuals. Gene pathways are annotated with different colors on the top panel of heatmap. Relative gene expression was calculated by normalizing the expression (TPM) of genes in each case to the average expression level (TPM) in control group, and were log10 transformed for plotting. The boxes corresponding to genes that differ significantly between severe cases and critically ill cases are highlighted with black borders. Genes that are significantly upregulated or downregulated in KTR patients compared to control individuals are marked with red upward triangles or blue downward triangles, respectively. Detailed expression data and differential expression analysis of these genes are provided in S13 Table.

To evaluate the immune status of these KTRs more precisely, we performed metatranscriptomic sequencing of whole-blood samples from 27 cases as described in a previous study [26], as well as the 14 healthy control individuals from that study (S6 Table). We analyzed 24,871 genes in total. Compared to the controls, 1,775 and 965 genes were significantly upregulated and downregulated in severe cases, respectively, while the equivalent numbers were 2,300 and 1,328 in the critically ill cases (S7-S9 Tables). The transcription levels of B cells, CD4 + T cells, CD8 + T cells were significantly lower in KTRs than controls (*p* < 0.001, *p* < 0.001, *p* < 0.001), and were also significantly lower in critically ill compared to severe cases (*p* = 0.006, *p* = 0.014, *p* = 0.001) (Fig 2A and 2B and S10 Table). An exception was patient Critically ill-3, a 23-year-old woman, who had a higher transcription level of T cells than all other KTRs and the most of controls. Gene Set Enrichment analyses (GSEA) of blood transcription modules (BTMs) [27] also revealed the dysfunction of both B cell- and T cell-related pathways (S2 Fig). Notably, the diversity and abundance of immunoglobulin (Ig)- and T-cell receptor (TCR)-related genes were all significantly lower in KTRs than controls (*p* < 0.001; *p* < 0.001; *p* < 0.001; *p* < 0.001), and the reductions were more pronounced in critically ill cases (Fig 2C and S11 Table).

With respect to innate immunity, X-cell analysis revealed that the transcription levels of activated myeloid dendritic cells (mDCs), M1 macrophages and neutrophils were significantly elevated in KTRs compared to controls (*p* = 0.001, *p* < 0.001, *p* = 0.029), but significant differences were not observed for monocytes (*p* = 0.539) (Fig 2A and 2B). Although there were no significant differences in the transcription levels of these four cells between the critically ill and severe groups, a high level of transcription was still apparent in some critically ill cases and even appeared concurrently in single cases. In contrast, the transcription of NK cells declined significantly in KTRs compared to controls (*p* < 0.001), which was also observed in critically ill compared to severe cases (*p* = 0.002).

Analyzing the transcription profile of the genes related to antimicrobial pathways in the GO database and cytokines revealed a significant upregulation of the 57 genes in KTRs (log2FC > 2.5, *p* adjust < 0.05) that are related to the interferon response against viruses and that encode neutrophil-related proteins, antimicrobial proteins against bacteria and fungi, and chemokines (Fig 2D and S12 and S13 Tables). Of the upregulated genes, the transcription levels of 15 genes including the HP gene were significantly higher in critically ill than severe cases. However, four genes were significantly downregulated, including IL7R, KLRK1, the Toll-like receptor TLR3, and the chemokine CXCL8. Taken together, the adaptive immune response was greatly suppressed in KTRs, although a partial innate immune response remained active.

#### Local immunity in KTRs.

**Fig 3:**
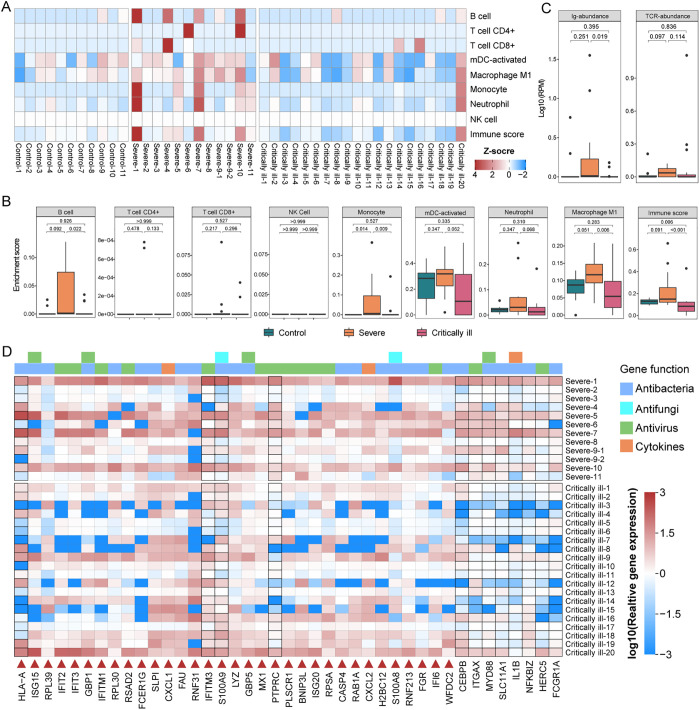
Local immune response in KTR cases with pneumonia. Cell type enrichment analysis of the BALF metatranscriptomes from KTR cases and healthy individuals using X‐Cell. The colors of the heatmap represent the Z‐scaled values (deviation from the mean across all samples), with warmer (red) colors indicating positive values (enrichment) and cooler (blue) colors indicating negative values (depletion) for each cell type. (B) Boxplots indicating the enrichment score of eight immune cell and the ‘immune score’ calculated by X‐Cell in BALF metatranscriptomes from healthy individuals, severe cases and critically ill cases. Differences between groups were estimated using a Mann-Whitney U-test. The horizontal box lines in the boxplots represent the first quartile, the median, and the third quartile. The whiskers cover all data points within 1.5 times the inter-quartile range and the black points denote outliers. Myeloid dendritic cell, mDC. (C) Boxplots indicating the abundance of Ig- and TCR-related genes in BALF metatranscriptomes from healthy individuals, severe cases and critically ill cases. Data are shown as natural log10 transformations for better visibility. (D) Heatmap indicating the relative expression of 41 significant differentially-expressed genes related to antimicrobial response pathways and the cytokine in BALF metatranscriptomes from KTR cases and control individuals. Gene pathways are annotated with different colors on the top panel of the heatmap. Relative gene expression was calculated by normalizing the expression (TPM) of genes in each case to the average expression level (TPM) in control group, and were log10 transformed for plotting. The boxes corresponding to genes that differ significantly between the severe cases and critically ill cases are highlighted with black borders. Genes that are significantly upregulated in KTR patients compared to control individuals are marked with red upward triangles. Detailed expression data and differential expression analysis of these genes are provided in S20 Table.

An analysis of BALF metatranscriptomes (32 KTR cases and 11 healthy individuals from our previous study [28]) revealed that the transcription level of B cells was extremely low (enrichment score <10^-16^) in the BALF samples from the most of critically ill cases (17/20) and controls (9/11) (Fig 3A and 3B and S14-S18 Tables), but higher (enrichment score >10^-3^) in severe cases (6/12). A significant difference was observed between severe and critically ill cases (*p* = 0.022), but not between the severe and controls (*p* = 0.092). The transcription levels of CD4 + T cells and CD8 + T cells in the BALF samples from KTRs were also very low, and generally comparable to those from controls, with the exception of six cases characterized by high transcription level of CD4 + T cells or CD8 + T cells. Although significant differences in mRNA abundance of Ig-related genes were observed between the severe and critically ill cases (*p* = 0.019), there were no significant differences between severe or critically ill cases and controls (Fig 3C and S19 Table). Furthermore, no significant changes in mRNA abundance of TCR-related genes were observed between any group.

In the case of innate immunity, the transcription levels of NK cells in all KTRs were very low but generally comparable to those in controls. The transcription levels of monocytes and M1 macrophages were significantly higher in severe than critically ill cases (*p* = 0.009, *p* = 0.006), while only slight differences were observed in the transcription levels of activated mDCs and neutrophils between them (*p* = 0.062, *p* = 0.068). However, with the exception of monocytes, which showed significantly higher transcription in severe cases than controls (*p* = 0.014), no significant changes in the transcription of other three cells appeared between severe or critically ill cases and controls. X-cell analysis of immune scores revealed a significantly higher level of immune infiltration in severe than critically ill cases (*p* < 0.001) as well as in controls (*p* = 0.091), but there was no significant difference between critically ill cases and controls (*p* = 0.006). Finally, higher immune infiltration was observed in three severe cases (Severe-1, Severe-7 and Severe-10), as well as Critically ill-20 who developed severe pneumonia just nine days post-transplant. This was consistent with the higher transcription levels of B cell and myeloid cells.

Finally, the transcription of 33 genes associated with interferon responses against viruses and involved in multiple pathways against bacteria and fungi, as well as the chemokines CXCL1 and CXCL2, were significantly upregulated in KTR cases compared to controls, and four genes were also significantly higher in severe than in critically ill cases (Fig 3D and S20 Table). In addition, other eight genes, related to regulation of immune responses (CEBPB, SLC11A1, HERC5), immune signaling (ITGAX, MYD88, NFKBIZ), antibody-dependent cellular phagocytosis (FCGR1A) and the proinflammatory cytokine IL1B, were significantly upregulated in severe compared to critically ill cases. In sum, the local adaptive immune response was also greatly suppressed in KTRs, while a partial local innate immune response remained active.

### Characterization of lung microorganism in KTRs

To characterize the active microorganisms in the lungs of these KTRs with pneumonia, we performed both metagenomic and metatranscriptomic sequencing on the BALF samples collected from each case, which generated a total of 6.86 and 5.09 billion reads, respectively (S21 Table). After removing low-quality and host genome reads, the valid reads ranged from 1.07 to 9.40 million in the DNA-seq libraries and from 1.02 to 61.18 million in the RNA-seq libraries. BALF samples collected from healthy individuals in a previous study [28] were used as controls and processed according to the same protocols (S22 Table).

#### Bacteria.

**Fig 4:**
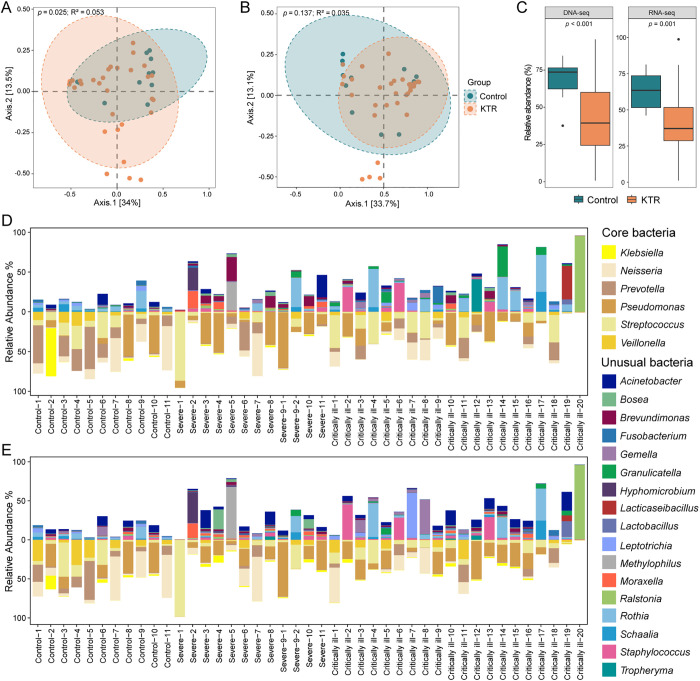
Comparison of lung bacterial microbiomes between KTR cases and healthy individuals. Principal coordinate analysis (PCoA) plot showing the composition of the active lung bacterial microbiome determined using BALF metagenomes (A) and metatranscriptomes (B) from each of the KTRs and healthy individuals. Beta-diversity was estimated by the Bray-Curtis dissimilarity index, and the Adonis (PERMANOVA) method was used to test the difference between KTR cases and healthy individuals. (C) Boxplots showing the total relative abundance of the core bacteria in the lung of KTRs and healthy individuals. Differences between groups were estimated using the Mann-Whitney U-test. The horizontal box lines in the boxplots represent the first quartile, the median, and the third quartile. The whiskers cover all data points within 1.5 times the inter-quartile range and the black points denote outliers. (D) and (E) Stacked barplots showing the relative abundance of core and unusual bacteria in each of KTRs and healthy individuals. The results were calculated based on the data derived from metagenomes (D) and metatranscriptomes (E) and presented separately. Core bacteria are stacked downwards from the X-axis, while the unusual bacteria are stacked upwards from the X-axis.

Bacteria with relative abundance ≥0.5% in both DNA-seq and RNA-seq libraries were considered to be “alive” in lungs. Those that might potentially represent background contaminants were removed. Consequently, a total of 56 bacterial genera were retained for subsequent analyses (S23 and S24 Tables). Comparisons of the lung bacteria in these KTRs to those in healthy individuals revealed no significant difference in alpha diversity by either the Simpson or Shannon indices based on either the DNA-Seq or the RNA-Seq data (S25 Table and S3 Fig). However, a principal coordinate analysis (PCoA) of metagenomes yielded a significant difference in beta diversity between these KTRs and healthy groups (*p* = 0.025; R^2^ = 0.053), although there was no significant difference in metatranscriptomes (*p* = 0.137; R^2^ = 0.035; Fig 4A and 4B). Hence, these data revealed no significant difference in species richness and evenness of lung bacterial communities between KTRs and controls, but there was a difference in bacterial composition and abundance.

Similar to previous studies [29,30], the most abundant bacteria in healthy individuals were *Prevotella*, *Neisseria*, *Streptococcus*, *Pseudomonas*, *Klebsiella* and *Veillonella*, accounting for 68.14% and 63.67% of the bacterial microbiome in metagenomes and metatranscriptomes, respectively. Hence, these six genera constituted the ‘core bacteria’ of lung microbiota in healthy individuals and are henceforth referred to in this manner. The abundance of these core bacteria declined significantly in KTRs in both metagenomes (40.63%, *p* < 0.001) and metatranscriptomes (40.69%, *p* = 0.001, Fig 4C), although there was no significant difference between severe and critically ill cases. However, the core bacteria in three patients (Severe-7, Critically ill-1 and Critically ill-11) were similar to those in healthy individuals. In addition, some of the core bacteria were at extremely high abundance (*Streptococcus* in patient Severe-1). Notably, in the most of KTRs, the core bacteria were replaced by 17 bacterial genera that were rare or absent in healthy individuals, including *Ralstonia*, *Methylophilus, Staphylococcus*, and *Tropheryma* (Fig 4D and 4E), which were referred to as ‘unusual bacteria’. The bacterial composition of the KTRs also varied considerably. For example, *Methylophilus* and *Ralstonia* dominated the microbiome in patients Severe-5 and Critically ill-20, respectively, but were at low abundance in other KTRs. Another interesting observation was that the bacterial composition in patient Severe-9 underwent markedly distinct changes during two episodes (Severe-9–1 and Severe-9–2) within a 10-month interval. Finally, for the remaining bacteria at low abundance, no significant differences were observed between KTRs and controls based on either metagenomes (*p* = 0.252) or metatranscriptomes (*p* = 0.924). Hence, these data reveal a large variation in the lung bacterial microbiome among KTRs and the lack of a distinct microbiological signature.

#### Viruses.

**Fig 5:**
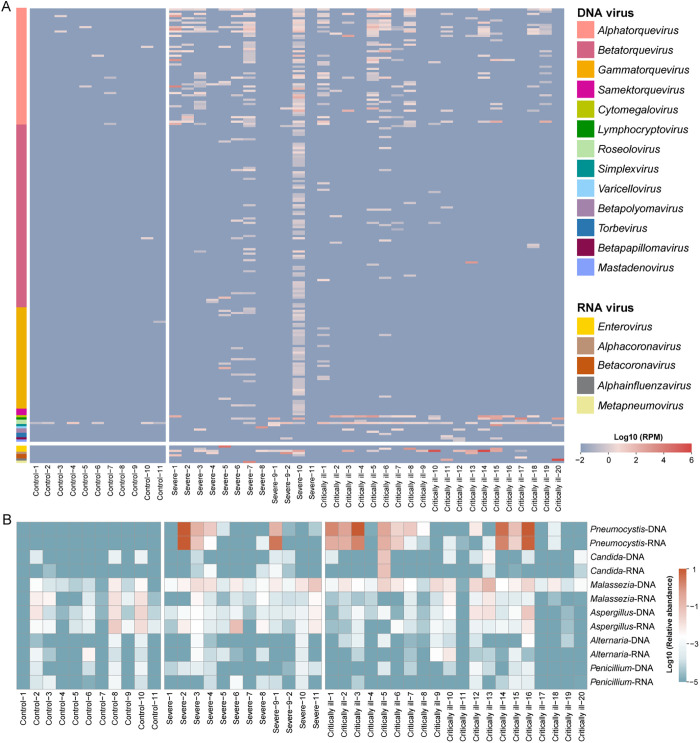
Comparison of the lung viral and fungal communities in KTR cases and healthy individuals. (A) Heatmap showing the relative abundance (RPM) of the viruses identified in the BALF samples from each of the KTRs and healthy individuals. All viruses were identified at the species level, and their genera were annotated with different colors on the left panel of the heatmap. The abundance of DNA viruses was estimated based on metagenomes, while the abundance of RNA viruses was derived from metatranscriptomes. The abundance (RPM) was log10 transformed for plotting. (B) Heatmap indicating the relative abundance of fungi in the BALF samples from each of KTR cases and healthy individuals. For each fungal genus, relative abundance was calculated based on metagenomic and metatranscriptomic data respectively. The relative abundance was log10 transformed for plotting.

197 species of DNA viruses belonging to 13 genera from six families were present in the lungs of these KTRs (Fig 5A and S26 and S27 Tables). Of the DNA viruses identified here, the most diverse and prevalent viruses were those from the family *Anelloviridae*, including 43 recognized species of four genera (*Alphatorquevirus*, *Betatorquevirus*, *Gammatorquevirus* and *Samektorquevirus*), and 142 tentative species according to the current criteria of taxonomy of this family [31,32]. These anelloviruses were found in 24 cases, with a substantial variation in the abundance of 0.11 to 3,511.21 reads per million (RPM). The number of viruses co-infecting single individuals ranged from two to 133. Other commonly-identified DNA viruses were human herpesvirus-7 (HHV-7, n = 21), cytomegalovirus (CMV, n = 13), Epstein-Barr virus (EBV, n = 9) and BK virus (BKV, n = 5), with abundance levels of ≥ 0.45, 0.48, 0.54 and 1.56 RPM, respectively. However, the remaining DNA viruses were detected in only one or two cases. In contrast, only HHV-7 and 11 species of anelloviruses were present in the BALF samples from healthy controls. Of note, anelloviruses were detected in six healthy individuals, but with low diversity and abundance (≤ 3 species; ≤ 1.85 RPM). HHV-7 was also detected in six healthy individuals, with abundances ≤ 1.85 RPM in five individuals and one case in which the abundance reached 18.31 RPM. Finally, the abundance and prevalence of CMV and HHV-7 were significantly higher in critically ill than severe cases (*p* = 0.007, *p* = 0.005; *p* = 0.004, *p* = 0.004).

Eight RNA viruses were identified in the BALF samples from these KTRs. In marked contrast, no RNA viruses were found in the controls. The most prevalent viruses were human rhinoviruses (HRV): human rhinovirus A (HRV-A, n = 1), human rhinovirus B (HRV-B, n = 4), and human rhinovirus C (HRV-C, n = 16). Five other viruses were identified in a few of cases, including human coronavirus-NL63 (HCoV-NL63, n = 4), human coronavirus-OC43 (HCoV-OC43, n = 2), SARS-CoV-2 (n = 1), influenza A virus (IAV, n = 2) and human metapneumovirus (HMPV, n = 1). The abundance of these RNA viruses ranged from 3.73 to 789,703.88 RPM (HRV-C in Critically ill-14). There was no significant difference in the prevalence of HRV between severe and critically ill cases.

#### Fungi.

Only six genera of fungi (*Alternaria*, *Aspergillus, Candida, Malassezia, Penicillium* and, *Pneumocystis*) were detected in KTRs, with ≥0.1% relative abundance (Fig 5B and S28 Table). Of these, *Alternaria*, *Aspergillus*, *Malassezia* and *Penicillium* were considered as likely background contamination due to their high abundance in the blank controls (see Methods). *Pneumocystis* had a higher prevalence in the critically ill (11/20) than the severe (4/12) cases, with abundance ranging from 84.23 to 229,733.37 RPM in metagenomes and from 33.46 to 113,494.39 RPM in metatranscriptomes, but was not found in the healthy individuals. Finally, although *Candida* was detected in nine KTR cases and three healthy individuals, a high abundance (563.20 RPM in metagenome and 580.53 RPM in metatranscriptome) was only found in one case (Critically ill-5) and was at much lower abundance in other cases (≤ 38.24 RPM in metagenomes and ≤ 13.25 RPM in metatranscriptomes).

### Determination and characterization of the agents involved in pneumonia

As all KTRs had severely suppressed immune systems, the microorganisms considered to be the likely etiologic agents of pneumonia included well-characterized pathogens, known opportunistic or accidental pathogens, and DNA viruses, bacteria and fungi that were at high abundance in KTRs but rare or absent in healthy individuals. The presence of these microorganisms in BALF samples from KTR patients was also validated by targeted PCR or RT-PCR. Consequently, 73 putative etiologic agents were documented, comprising 46 DNA viruses, 17 bacteria, eight RNA viruses, and two fungi (Table 1, S29 and S30).

**Table 1:** Etiological agents identified in BALF samples from 32 KTR cases with pneumonia.

Patient	Bacteria	Fungi	DNA virus	RNA virus
Severe-1	*S. pneumoniae*	–	Anelloviruses, CMV	HRV-C, HCoV OC43
Severe-2	*H. nitrativorans, F. osloensis*	*P. jirovecii*	Anelloviruses	–
Severe-3	–	*P. jirovecii*	Anelloviruses, BKV	–
Severe-4	–	*P. jirovecii*	–	–
Severe-5	*Methylophilus sp. TWE2*	–	Anelloviruses	HRV-A
Severe-6	–	–	Anelloviruses	–
Severe-7	–	–	Anelloviruses	HMPV
Severe-8	–	–	VZV	–
Severe-9–1	*P. aeruginosa*	*P. jirovecii*	EBV, HSV-1	–
Severe-9–2	–	–	HHV-7	HCoV NL63
Severe-10	–	–	Anelloviruses, EBV	HRV-C
Severe-11	–	–	–	–
Critically ill-1	–	*P. jirovecii*	Anelloviruses, HHV-7	–
Critically ill-2	S. epidermidis	*P. jirovecii*	Anelloviruses, CMV	–
Critically ill-3	–	*P. jirovecii*	Anelloviruses, CMV, HHV-7, BKV	HRV-C
Critically ill-4	*R. mucilaginosa*	–	CMV	–
Critically ill-5	–	*P. jirovecii,* *C. albicans*	Anelloviruses, CMV, JCV	–
Critically ill-6	*S. epidermidis*	*P. jirovecii*	Anelloviruses, CMV, HuLaBV	HRV-B
Critically ill-7	*Leptotrichia sp. oral taxon 212*	*P. jirovecii*	CMV, BKV	–
Critically ill-8	*Gemella sp. oral taxon 928*	*P. jirovecii*	Anelloviruses, CMV	HRV-B
Critically ill-9	–	–	–	–
Critically ill-10	–	–	EBV	HRV-C
Critically ill-11	–	–	HuLaVV	IAV
Critically ill-12	*T. whipplei*	–	BKV	HRV-C
Critically ill-13	*S. hominis*	–	Anelloviruses	HRV-C, HCoV NL63
Critically ill-14	*R. mucilaginosa, G. adiacens*	*P. jirovecii*	Anelloviruses, CMV, EBV, BKV	HRV-C
Critically ill-15	–	*P. jirovecii*	Anelloviruses, CMV, EBV, HSV-1	HRV-B, HRV-C
Critically ill-16	–	*P. jirovecii*	CMV	–
Critically ill-17	*R. mucilaginosa,* *S. odontolytica*	–	EBV	SARS-CoV-2
Critically ill-18	–	*P. jirovecii*	Anelloviruses, CMV, HHV-7	–
Critically ill-19	*Lactobacillus sp. JM1,* *L. paracasei, M. tuberculosis*	–	Anelloviruses	–
Critically ill-20	*R. mannitolilytica*	–	EBV	IAV

Abbreviations: *S. pneumoniae, Streptococcus pneumoniae; H. nitrativorans, Hyphomicrobium nitrativorans; F. osloensis, Faucicola osloensis; P. aeruginosa, Pseudomonas aeruginosa; S. epidermidis, Staphylococcus epidermidis; R. mucilaginosa, Rothia mucilaginosa; T. whipplei, Tropheryma whipplei; S. hominis, Staphylococcus hominis; G. adiacens, Granulicatella adiacens; S. odontolytica, Schaalia odontolytica; L. paracasei, Lacticaseibacillus paracasei; M. tuberculosis, Mycobacterium tuberculosis; R. mannitolilytica, Ralstonia mannitolilytica. C. albicans, Candida albicans; P. jirovecii, Pneumocystis jirovecii.* BKV, BK polyomavirus; HCMV, Human Cytomegalovirus; HHV-7, Human Herpesvirus 7; HSV-1, Herpes Simplex Virus 1; VZV, Varicella-Zoster virus; EBV, Epstein-Barr virus; JCV, JC polyomavirus; HuLaBV, human lung-associated brisavirus; HuLaVV, human lung-associated vientovirus. HRV-A, Human Rhinovirus-A; HRV-B, Human Rhinovirus-B; HRV-C, Human Rhinovirus-C; HCoV, Human coronavirus; IAV, Influenza A virus; HMPV, Human Metapneumovirus.

All viruses belonging to the family *Anelloviridae* were categorized under “Anelloviruses” in this table, and the details of their species and abundance are provided in S29 Table.

The likely bacterial pathogens comprised 17 species. Of these, 15 are known to cause pneumonia, while the remaining two – *Hyphomicrobium nitrativorans* and *Methylophilus sp. TWE2* – have not been reported to be associated with pneumonia. *Rothia mucilaginosa* and *Staphylococcus epidermidis* were identified in three and two cases, respectively, while the remaining 15 bacteria were each found in one case. Phylogenetic analysis revealed that these bacteria were closely related to those present widely in and outside of China (S4 Fig).

Eight RNA viruses and 46 DNA viruses were determined as likely etiological agents. The DNA viruses comprised 37 species of anellovirus, EBV, HCMV, HHV-7, HSV-1, VZV, BKV, JCV, human lung-associated vientovirus (HuLaVV) and human lung-associated brisavirus (HuLaBV). Their abundance ranged from 1.10 to 4105.26 RPM in metagenomes, but was relatively low in metatranscriptomes, ranging from 0.00 to 2835.68 RPM, likely reflecting the presence of relatively few cells in the BALF samples. Although there is no consensus on the pathogenicity of anelloviruses in humans [33], we considered anelloviruses with abundance values > 3.69 RPM to be involved in lung infections*.* Of the DNA viruses, anelloviruses and HCMV were most common and associated with 24 and 12 cases, respectively, followed by EBV (n = 7) and BKV (n = 5) and HHV-7 (n = 4). Remarkably, co-infection by two to 30 species of anelloviruses occurred in 19 cases. In contrast, the remaining five viruses each were only responsible for one or two cases. In addition, EBV, HCMV and anelloviruses were also found in the blood samples from five, five, and 20 cases, respectively (S31 Table). Finally, all DNA viruses identified here were closely related to known viruses recovered from humans in and outside of China (S4 Fig).

RNA viruses were likely responsible for disease in 15 KTRs, with their abundance ranging from 3.70 to 789,703.90 RPM. Of these, three species of HRV were associated with 11 cases, including the co-infection of HRV-B and HRV-C in patient Critically ill-15. Five other RNA viruses (IAV, HMPV, HCoV-NL63, HCoV-OC43 and SARS-CoV-2) were likely responsible for seven cases. Phylogenetic analysis revealed that the HRVs grouped into six subtypes, sharing close relationship with known corresponding viruses that are widely prevalent in and outside of China, while the other RNA viruses identified here were also closely related to known viruses (S5 Fig).

Similar to previous findings [33], we identified human pegivirus (HPgV) in blood samples from 12 cases, although it was at relatively high abundance in three (7.52-1811.82 RPM) and very low (≤3.00 RPM) in the remaining cases (S31 Table). Due to its absence in BALF sample, HPgV was not considered to be associated with lung infections. For the three hepatitis B patients, only two reads were detected in the blood metatranscriptome of Severe-5, probably due to the use of long-term antiviral therapy.

The fungal agents identified in KTRs included *P. jirovecii* and *C. albicans*. A high abundance of *P. jirovecii* was identified in 15 (46.88%) cases, with multi-locus sequence typing revealing five genotypes in the KTRs. The co-presence of two or more genotypes in single individuals was observed in seven cases (S32 Table). In contrast, a high abundance of *C. albicans* was only observed in patient Critically ill-5, which was closely related to those circulating in and outside of China (S4 Fig).

Finally, although two or more agents were found in 27 cases, with all four groups of pathogens identified in four cases, only a single pathogen was identified in three cases (Table 1) and none were identified in two cases (Severe-11 and Critically ill-9). The latter may reflect the delay in BALF sampling (12 days after admission for Severe-11) or the use of antimicrobial therapy.

## Discussion

Immune defenses generated inside and outside of lung are crucial to protect hosts against pneumonia [4]. However, for KTRs and other SOT recipients, immunosuppressants received for prolonged graft survival dampen their immune function [34]. Although nearly three-quarters of the KTRs studied here ceased to take immunosuppressants, or took them at a reduced dose, their extrapulmonary immunity was impaired severely, similar to previous studies [35–38]. Blood tests revealed the dysfunction of their adaptive immunity, including the dramatic reduction of lymphocytes. The elevation of NK cells, monocytes and neutrophils was only observed in a few of cases. Importantly, the transcription levels of B cells, CD4 + T cells, CD8 + T cells were significantly decreased in KTRs, particularly in critically ill patients. In addition to the disruption of B-cell and T-cell related pathways, both the mRNA diversity and the abundance of the Ig- and TCR-related genes decreased significantly in KTRs, and this reduction was most pronounced in critically ill cases. Finally, the significant upregulation of over a dozen of genes in critically ill cases, some of which are associated with inflammation [39,40], may partially explain why their associated clinical symptoms are more severe.

Local immunity plays a key role in the fight against lung infections [4,41]. In the face of commensals and potential pathogens within lungs, it is necessary for the host immune system to sense and tolerate non-pathogenic commensal and to purge resident pathogens and new invaders to prevent damage to lungs from inflammatory reactions [8]. Although animal experiments have revealed that calcineurin impairs local lung immunity [42,43], far less is known about the lung immune status in KTRs with pneumonia. The transcription level of B cells in the BALF samples from the most of critically ill cases analyzed here was extremely low. Although the transcription level in severe cases was significantly higher than in critically ill cases, there was no significant difference between severe cases and controls. A similar phenomenon was observed in the abundance of Ig-related genes. Notably, the mRNA abundance of TCR-related genes in both severe and critically ill cases was comparable to that in controls. In the case of innate immunity, the transcription levels of activated mDCs, macrophage M1, and neutrophils were extremely low in all or the most of these KTRs, and generally comparable to those in controls. Transcription levels were not elevated in all KTRs for NK cells, and increased significantly only for monocytes in severe cases. For genes related to antimicrobial responses and cytokines, the number that were significantly-upregulated was greater in severe cases than in critically ill cases. From a functional viewpoint, these genes were primarily associated with the interferon system, direct antibacterial activity, as well as chemokines responsible for recruiting inflammatory cells. Hence, these data indicate that lung immunity was also impaired severely in these KTRs, especially in critically ill cases.

Commensal microorganisms of the lung are believed to act as gatekeepers that provide resistance to colonization by respiratory pathogens [8,15]. Alteration in the lung microbiome has been observed in pneumonia and other lung diseases, characterized by a reduction in alpha diversity and an enrichment of opportunistic pathogens [7,9]. Although the lung microbiome also varies among disease states [44], distinct bacterial signatures are often associated with different lung diseases [9,10,17,45]. To date, however, the association between the lung microbiome and respiratory infections in KTRs and other SOT patients remains unknown. The metagenomic and metatranscriptomic data generated here revealed a high diversity of active bacteria in KTRs. Remarkably, unlike the reduction in immunocompetent persons with pneumonia [8,9], alpha diversity in the KTRs was similar to that of healthy individuals. However, similar to previous reports in other immunocompromised patients [46,47], beta diversity varied significantly in KTRs. Importantly, the abundance of the core bacteria decreased in KTRs, and was replaced by 17 genera of bacteria present in upper respiratory tract (e.g., *Leptotrichia*, *Rothia*, *Gemella*) or from environments (e.g., *Methylophilus*, *Hyphomicrobium*). Remarkably, bacterial composition and abundance varied considerably among the KTRs and even between the two disease episodes in patient Severe-9. Hence, these data indicate that the lung bacterial microbiome in KTRs with pneumonia is enriched with a high diversity of unusual bacteria and exhibits extensive variation without a common microbial signature.

An extensive diversity of DNA viruses and RNA viruses was present in the lung microbiome of the KTRs studied here. Previous studies have revealed the presence of multiple DNA viruses, including anelloviruses and herpesviruses in blood and other samples from healthy individuals [48,49] as well as immunocompromised individuals [33,50]. However, only a small number of viruses have been identified in the lung [30], and the atlas of lung virome in either healthy or immunocompromised individuals remains unclear. Herein, we documented an extremely high diversity of DNA viruses in BALF samples from KTRs, in marked contrast to much lower levels in BALF samples from healthy individuals, while RNA viruses were only found in the KTRs and not in the controls. Remarkably, 43 defined and 142 tentative anelloviruses were identified, which raises the question of from where such highly diverse and abundant anelloviruses originated? Finally, compared to bacteria and viruses, the diversity and the abundance of fungi were lower in KTRs and healthy individuals, supporting the idea that most fungi cannot grow in the human internal environment [51].

Due to the complex complications and the clinical atypicality of pneumonia in KTRs [1,24,52], arriving at a specific microbiologic diagnosis has presented a considerable challenge [21]. Through a combination of metagenomic and metatranscriptomic approaches we provide a definitive microbiologic diagnosis in 28 KTR cases. A total of 73 agents were identified and characterized from BALF samples, including 17 bacteria, two fungi and 54 viruses. Notably, the majority of these cases (84.38%) were characterized by multiple co-infections, including the co-presentation of bacterial, fungal, DNA and RNA viral agents in three cases, which confused routine microbial screening at the clinical laboratory of the hospital (S33 Table). In addition to well-known opportunistic infections, bacterial pathogens such as *S. pneumoniae* and *P. aeruginosa*, 15 unusual bacteria (i.e., that are accidental infections or absent in healthy individuals) were found in lung infections. Of note, two of these have not previously been reported to be associated with pneumonia (S30 Table). Similarly, although the association between anelloviruses and specific diseases remains unproven [33], the extremely high abundance of these viruses in the lungs of the KTRs studied here, especially compared to healthy individuals, suggests that they are directly or indirectly involved in pneumonia, perhaps in a similar manner of opportunistic bacteria agents. Further studies on anellovirus pathogenicity are clearly required. Finally, some of bacterial agents involved in pneumonia were not detectable in this study, likely due to the use of antibiotics before admission.

Immune defenses generated in and outside of lung are crucial to protect against infection [4], while the dynamic and balanced lung microbiome plays an important role in the maturation and maintenance of homeostasis of respiratory physiology and immunity, as well as the prevention of diseases [8–12]. The complex interplay between the microbiome and host immunity also affects the progression and clinical manifestations of disease [9]. However, for KTRs with pneumonia, impaired immunity resulted in a substantial alteration in their lung microbiome, with the colonization or even overgrowth of unusual microorganisms, and the significant loss of core commensal bacteria, which might in turn hinder the effective clearance of pathogens, lead to co-infections, and increase disease severity.

In sum, the data generated here suggest that immune dysfunction in and outside of lungs enabled opportunistic, rare and accident microorganisms to prosper, and allowed the overgrowth of latent viruses or the growth of new invaders in the lungs of KTR patients, leading to both multiple co-infections and a major change in the lung microbiota. The combination of multiple co-infections (involving bacteria, fungi, and viruses) and an immunocompromised host was in turn associated with worse disease outcomes.

## Materials and methods

### Ethics statement

This study was approved by the Ethics Committee of Zhongshan Hospital, Shanghai (permit number: Zhongshan Hospital Ethics Committee B2019-112R2). Written informed consents were obtained from all patients or their relatives. All samples were collected from the patients as a part of routine clinical testing.

### Study design and patient cohort

We enrolled KTR patients clinically diagnosed with pneumonia by CT scan and hospitalized at Zhongshan Hospital in Shanghai, China during Feb 28^th^ 2019 to January 16^th^ 2025. The demographic and hospital admission logs were obtained from electronic hospital information system. Initial laboratory tests conducted upon admission of all patients, including blood counts, biochemical markers, immune cells, and cytokines, were performed at the Clinical Laboratory of Zhongshan Hospital. Additionally, their chest CT images were independently reviewed by three professional radiologists. Relevant data, including kidney disease before transplantation, intervals from transplantation to the admission, and immunosuppressive therapy, were also obtained.

### Sample collection

BALF and whole blood samples were collected from the KTR cases. Two 5 mL blood samples were collected for clinical routine tests and microbial and immune assays. For BALF samples, bronchoscopy was performed by professional pulmonary physicians within 72 hours following admission or upon diagnosis following a standardized protocol, with 10 mL of instilling sterile saline solution for four times (40 mL in total) into distal bronchial trees. Both blood and BALF samples for microbial and immune tests were immediately stored at −80°C until usage.

### DNA-Seq and RNA-Seq library preparation

Both metagenomic and metatranscriptomic sequencing of BALF samples were used to determine the lung microbiome, identify likely pathogens, and determine local immune responses according to the standard protocols established in our laboratory [3,26,28]. Briefly, total RNA was extracted from 200 μL of BALF or blood using the RNeasy Plus Universal Mini Kit (*Qiagen*, Hilden, Germany). For BALF samples, after quality control, the libraries were constructed using the SMARTer Stranded Total RNA-seq Kit v2 - Pico Input Mammalian (*Takara Bio*, Shiga, Japan). For metagenomic library preparation, total DNA was first extracted from a 400 μL BALF sample using the FastPure Microbiome DNA Isolation Kit (*Vazyme*, Nanjing, China), and further quantified by a Qubit4.0 fluorometer (*Invitrogen*). Subsequently, libraries were constructed using the VAHTS Universal Plus DNA Library Prep Kit for Illumina V2 (*Vazyme*, Nanjing, China) following the standard protocol. In addition, library preparation of enzyme-free water was performed using same approach, generating two and one “blank” DNA and RNA library controls, respectively. For blood samples, RNA library construction was performed using the VAHTS Universal V10 RNA‐seq Library Prep Kit for Illumina sequencing (*Vazyme* Nanjing, China). Ribosomal and globin RNAs were depleted using the Ribo-off Globin & rRNA Depletion Kit (*Vazyme*, Nanjing, China). All libraries were sequenced on an Illumina Novaseq 6000 or Novaseq X plus platform in 150 bp paired-end manner by the *Novogene* Bioinformatics Institute (*Novogene*, Beijing, China) after quantification.

### Analyses of differentially expressed genes in blood and BALF

The initial processing of metatranscriptomic raw reads, including adapter and quality trimming, was performed as described in our previous studies [28,53]. Clean data from the BALF and blood transcriptomes were used for the analysis of gene expression. Whole blood metatranscriptomes from 14 healthy individuals [26], as well as those generated herein from the BALF samples collected from 11 healthy individuals in our previous study [28], were used as healthy controls. Sequence reads were first aligned to the human reference genome (GRCh38.p13) using the HISAT2 software (version 2.2.1) [54]. Samtools (version 1.19.2, https://github.com/samtools/samtools) was then used to generate intermediate files for quality assessment via BamQC (version 2.3, https://github.com/s-andrews/BamQC) and for analyzing the insertion size distribution. Next, the featureCounts tool within the subread package (version 2.0.6) [55] was employed to determine gene exon read counts. After excluding mitochondrial genes, a total of 60,606 genes were retained (version: Homo_sapiens.GRCh38.p13.gtf). Finally, the gene expression matrix was generated using the genes with a read count of at least 10 in at least two libraries, and was length-normalized as the number of Transcripts Per Million (TPM).

Prior to downstream analysis, a comprehensive QC analysis was performed and all libraries passed (≥ one million reads, ≥ 8000 expressed genes with TPM ≥ 0.5). The DESeq2 package (version 1.44.0, https://github.com/mikelove/DESeq2) was subsequently applied to identify differentially expressed genes (DEGs), with statistical significance set at *p* adjust ≤ 0.01 (corrected by the Benjamini-Hochberg method) and a log2foldchange ≥ 2 or ≤ -2 for upregulation and downregulation, respectively. GO and KEGG analyses were performed with R package *clusterProfiler* v4.12.0 based on DEGs calculated above.

### Analysis of immune responses

The analysis of immune responses was mainly based on the methods established previously in our laboratory, with minor modifications [26]. To infer the immune repertoire, Ig- and TCR-related genes were extracted from clean BALF and blood RNA-seq data using MiXCR [56,57]. VDJtools [58] was then used to compute the number of clonotypes. Notably, since the rarefaction curves for both IG and TCR clonotypes in the BALF RNA-seq library failed to reach a plateau, the diversity estimation was excluded from further analysis.

The enrichment scores of various immune cell types in both blood and BALF were analyzed using the X-Cell algorithm [59] from the R package *immunedeconv* [60], and based on the expression profile (calculated by TPM) of all samples, which served as raw signatures. All cell enrichment scores were normalized using Z-scores for better visualization in heatmaps. In addition, GSEA was performed to identify significantly-enriched gene sets correlated with BTMs [27]. Briefly, the DESeq2 results comparing critically ill and severe cases to healthy controls were used as raw input, and the GSEA was performed using the R package *clusterProfiler*, with the Normalized Enrichment Score (NES) used to show enrichment level.

To analyze the expression of anti-microbial and cytokine genes, we constructed three gene sets from GO (defense responses to virus, responses to bacterium, and defense responses to fungi) and a compilation of 128 cytokine genes (see S12 Table). Of note, only genes with a log2fold change ≥2.5 or ≤ -2.5 and an adjusted *p*-value ≤ 0.05 were considered as evidence of significant differential-expression. The relative expression of each gene was calculated as the ratio of the TPM in each patient to the average TPM of healthy individuals.

### Analysis of lung microbiomes

For metagenomic raw reads, data quality was first assessed using FastQC (version 0.12.1) (http://www.bioinformatics.babraham.ac.uk/projects/fastqc/). Adapter sequences and duplicated reads were removed using fastp (version 0.23.4) [61]. Paired-end reads were merged, followed by the removal of low-complexity sequences using bbmap (version 38.84.0, https://github.com/BioInfoTools/BBMap). All clean reads obtained from metagenomics and metatranscriptomics were aligned to the human genome (GCF_000001405.39_GRCh38.p13) using bowtie2 (version 2.5.2) [62] with the “--very-sensitive” parameter employed to exclude human-related sequences. The ‘no-human reads’ were then used for microbiome analysis.

#### (A) Bacteria.

We used Kraken2 (version 2.1.3) and Bracken (version 2.9) [63,64] to determine bacterial taxa and their abundance in the sequence data, with all ‘no-human reads’ aligned to the PlusPF database (https://benlangmead.github.io/aws-indexes/k2). Results assigned to “Bacteria” and “Archaea” at the genus level were extracted, and the relative abundance of each genus was initially calculated as the percentage of its reads relative to the total number of prokaryotic reads. Given that we focused on the bacteria that were considered “alive” in lung, a relatively stringent threshold was applied. Specifically, only genera with a relative abundance ≥ 0.5% in both the DNA-seq and RNA-seq libraries of one sample were included in subsequent analyses.

Considering the low biomass of BALF samples, a contamination exclusion protocol was established to eliminate background contamination during nucleic acid extraction and library construction. Based on the matrix of DNA-Seq and RNA-Seq read counts for all bacteria, correlations between individual bacterial genera were computed using the “cor()” function in R. Accordingly, the following bacteria were considered to be contaminants: (i) genera which did not belong to common lung bacteria [9], but were the top 10 most abundant genera in the blank controls, and (ii) genera which did not belong to common lung bacteria but had a correlation coefficient > 0.9 with genera belonging to those in category (i). All the microorganisms found in the blank controls and their relative abundance are listed in S34 Table, while the bacterial genera excluded during this process are listed in S35 Table. Finally, the alpha diversity of lung bacteria was calculated based on definitions of the Shannon and Simpson indices. Beta diversity was explored using the R package *phyloseq* (version 1.48.0) [65]. Bray-Curtis dissimilarity was used as the dissimilarity measure, with visualizations performed using PCoA.

#### (B) DNA viruses and fungi.

As the PlusPF database does not include the fungi like *P. jirovecii* and has not been updated with recent DNA virus taxa, Kraken2 was not used in their analysis. Instead, we employed the following procedure. We first removed the reads previously assigned to bacteria, archaea, and *Homo sapiens* from the ‘nohuman reads’ set in the metagenomic data, which yielded a database of ‘no human-no bacteria-reads’ (NHNB-reads for short). The remaining reads were compared to the non-redundant nucleotide databases using blastn (version 2.9.0) [66]. The resulting sequence alignments were then filtered according to the following thresholds, with the following sequences removed: (i) reads with alignment lengths <75 bp; (ii) reads not belonging to DNA viruses or fungi; (iii) reads belonging to bacteriophages, plant DNA viruses, as well as non-human animal DNA viruses which subsequently proved to be contaminants or result from bioinformatic errors.

We used both relative and absolute abundance to delineate microbial abundance. Given that bacteria were present in the lungs of all patients, we defined their relative abundance as the ratio of the read counts for each agent to the total read counts of all bacteria. Absolute abundance, which better reflects the proportion of a specific agent within the entire microbial community, was normalized to RPM and computed as follows: read count of each agent/total ‘non-human reads’ × 1,000,000.

For fungi, we first counted the reads of each fungus at the genus level and then calculated the relative abundance of each fungal genus as we did for bacteria. Only genera with relative abundance >0.1% were considered “alive”. As the smaller genomes of DNA viruses relative to bacteria and fungi might lead to low read counts, no additional filtering was performed. We also calculated the abundance of these DNA viruses and fungi in the RNA-seq libraries. Specifically, the reference genomes of all target fungi and DNA viruses from blastn were retrieved and used as a template for read mapping by bowtie2. The ‘NHNB-reads’ in the RNA-seq libraries were then mapped to these templates. All mapped reads were further matched against nucleotide database using blastn to exclude error mapping.

As the taxonomic classification of the family *Anelloviridae* in the nucleotide database lags behind that of the ICTV, anelloviruses in the BALF samples were defined according to the ICTV criteria for this family (https://ictv.global/taxonomy) [31,32]. All sequences belonging to 173 species of 34 genera of *Anelloviridae* were retrieved from the NCBI Virus database (https://www.ncbi.nlm.nih.gov/labs/virus/vssi/), and only those labeled as ‘complete genome’ with a length ≥ 2,000 nucleotides (n = 5374) were used. All sequences were initially processed using ORFfinder (version: 0.4.3, https://www.ncbi.nlm.nih.gov/orffinder/) to identify the ORF1 gene of anelloviruses. Subsequently, cd-hit (version: 4.6, http://www.bioinformatics.org/cd-hit/) was used to cluster the ORF1 gene sequences obtained based on a threshold of 69% similarity, resulting in a total of 807 clusters. Among these, 173 species defined by the ICTV were each assigned to distinct clusters, confirming the accuracy and robustness of this method. The remaining clusters were considered as tentative species. For the assignment of genera, we constructed a maximum likelihood phylogenetic tree based on the ORF1 gene. The genus assignment of each tentative species was determined according to their phylogenetic position [31]. Finally, we constructed an index including all 5374 complete genome sequences and 173 reference sequences defined by the ICTV. Using blastn, the corresponding species for these reads were determined based on blasting against this index.

#### (C) RNA viruses.

RNA viruses were analyzed based on the protocol previously established in our laboratory [3,28,53]. Briefly, ‘NHNB-reads’ from each metatranscriptome library were assembled *de novo* using Trinity (version 2.15.1) [67] with default parameters. Subsequently, all assembled contigs were matched against the nucleotide database using blastn, and then against the non-redundant (nr) protein database with Diamond Blastx (version 0.9.24.125) [68]. We then calculated the abundance of RNA viruses by constructing an index based on the genomes of the closest related complete RNA viral genomes from the BLAST results and mapping the ‘NHNB-reads’ from the metatranscriptome to this index using Bowtie2. Finally, the reads mapped to this index were further matched against the nucleotide database using blastn to exclude error mapping.

### Determination of agents associated with pneumonia

In addition to its virulence, to determine whether a particular microorganism was involved in lung infections we considered its abundance in KTRs relative to healthy individuals [69,70]. For bacteria, those that met the following criteria in both metagenomic and metatranscriptomic data were considered as likely causative agents: (i) unusual bacteria with a relative abundance greater than 20% in metagenomes or metatranscriptomes, and ≥ 1.5-fold higher than that of the metagenomes and metatranscriptomes in healthy controls; (ii) core bacteria with a relative abundance >60% in both metagenomes and metatranscriptomes. In addition, due to the difficulty of DNA extraction from *Mycobacterium tuberculosis* [71], it was considered as likely causative agents when at least 10 reads were assigned to this species by Karken2. Finally, the presence of their DNA and RNA in BALF samples was confirmed by PCR or RT-PCR assays using specific primers designed based on their species.

The abundance of DNA viruses was extremely low in BALF metatranscriptomes. As they were also present in the metagenomes of healthy individuals, only DNA viruses in KTRs with an abundance ≥ 2-fold higher than healthy controls were defined as the agent involved in lung infections. In addition, viruses that were absent in healthy controls and had an abundance >1.0 RPM in the BALF metagenomes from KTRs were considered as likely causative agents. All identified agents were confirmed by PCR assays. In addition, we performed qPCR and RT-qPCR to quantify the viral loads and transcriptional activity of anelloviruses, CMV, EBV, and HHV-7. Notably, RNA viruses were only detected in KTR BALF samples and belonged to well-known respiratory pathogens. Their presence was also confirmed by RT-PCR assays to exclude cross-contamination caused by index-hopping.

For fungi, only *C. albicans* and *P. jirovecii* were at high abundance in KTRs. Their DNA and RNA in BALF were also confirmed by PCR or RT-PCR. Hence, these two fungi were defined as causing lung infections.

We also used PCR or RT-PCR to determine whether all agents identified in the BALF samples were also present in blood samples, and performed a similar analysis for the whole-blood metatranscriptomes to identify the agents in blood.

### PCR and qPCR

PCR and RT-PCR were employed to confirm the presence of the causative agents determined by DNA-Seq and RNA-Seq, and to amplify the sequence fragments needed for phylogenetic analysis. For all PCR and RT-PCR assays, the Takara One Step Prime Script III RT-PCR kit (*Takara Bio*, Shiga, Japan) or 2 × AceTaq Master Mix (*Vazyme*, Nanjing, China) was used according to the standard protocol. Subsequently, all PCR products were visualized by agarose gel electrophoresis and sent to Sheng Gong Biotechnology Co., Ltd (Shanghai, China) for Sanger sequencing. The sequencing results were inspected and assembled in Geneious Prime (version 2020.1.2) (https://www.geneious.com), followed by BLAST analysis against the GenBank database (https://blast.ncbi.nlm.nih.gov/) for species identification and sequence similarity assessment. For qPCR and RT-qPCR, the HiScript II One Step qRT-PCR Probe Kit (Takara Bio, Shiga, Japan) or the AceQ Universal U+ Probe Master Mix (Vazyme, Nanjing, China) was used according to the standard protocol. All primer sequences used in this study are provided in S36 Table. All the complete or partial genomes of the infectious agents recovered in this study and used for phylogenetic analysis are provided in S37 Table.

### Phylogenetic analysis

Phylogenetic analysis was performed based on whole genome or representative gene sequences of the pathogens. The reference data set for each phylogeny included sequences that were representative of the background genetic diversity for each of the infectious agents as well as the closest hits in the blastn analysis. All sequences recovered here and reference sequences were aligned using the FFT-NS-2 algorithm implemented in MAFFT (version 7) [72], and ambiguously aligned regions with missing data were removed using Trimal (version 1.2) [73]. Maximum likelihood phylogenetic trees for each of the infectious agents were then estimated in IQ-TREE2 [74] with 1,000 bootstraps and using the parameter “-m MFP” to determine the best-fit substitution model. All trees were visualized using FigTree (version 1.4.4) (http://tree.bio.ed.ac.uk/software/figtree/).

### Statistical analysis

Categorical variables were displayed as counts or percentages, and Fisher’s exact tests were used for their comparison. Continuous variables were compared using Mann–Whitney U tests. *P*‐values were adjusted using a Bonferroni correction or the Benjamini and Hochberg False Discovery Rate (FDR) in multiple comparisons. The Adonis (PERMANOVA) method in the R package *pairwiseAdonis* was used to test the statistical significance of the Bray-Curtis PCoA. All analyses were performed using SPSS version 20.0 (SPSS, Chicago, IL, USA) or R software (https://www.r-project.org/), and *p* < 0.05 was considered as a significant difference at any condition. Finally, Graphs were generated in R software, including the R package *ggplot2*, *pheatmap* and *phyloseq*. All figures were prepared and modified using Adobe Illustrator CS6 (Adobe Systems, San José, USA).

## Supporting information

S1 Fig Chest computed tomography (CT) of all 32 KTR cases. For each case, the most representative CT image on the day of admission is provided.
(TIF)

S2 Fig Transcriptional profiles reflecting the systemic immune response in KTR cases. Gene Set Enrichment analyses (FDR < 0.25; 1,000 permutations) were used to identify positive (red), negative (blue), or no enrichment of immune BTMs (gene sets). The color of each point shows the normalized enrichment score (NES) of each BTM selected, and the size of each point shows the *p* value.
(TIF)

S3 Fig Comparison of alpha diversity in lung bacterial microbiomes from KTR cases and healthy individuals. Boxplots showing the Shannon and Simpson indices of the lung bacterial microbiome in KTRs and healthy individuals. These indices were calculated based on BALF metagenome and metatranscriptome data. Differences between groups were tested using a Mann-Whitney U-test. The horizontal box lines in the boxplots represent the first quartile, the median, and the third quartile. The whiskers cover all data points within 1.5 times the inter-quartile range and the black points denote outliers.
(TIF)

S4 Fig Phylogenetic analyses of bacterial, fungal and DNA viral agents identified in this study. **(A)** Maximum likelihood phylogenetic tree based on the 16s rRNA gene of the bacterial agents identified here and reference strains from GenBank. **(B)** Phylogenetic tree based on the cytochrome b gene of the fungi identified here and reference strains from GenBank. **(C)** Phylogenetic trees based on representative genes of the DNA viruses identified here and reference strains from GenBank. Sequences identified in this study are marked with red bold characters. Bootstrap values (> 70%) are shown at the branch nodes. For larger trees, only the lineages or sub-lineages that contain sequences identified in this study are provided.
(TIF)

S5 Fig Phylogenetic analyses of RNA viruses identified in this study. Maximum likelihood phylogenetic trees were estimated based on the complete genome or representative genes of the RNA viruses identified herein. The sequences recovered in this study are marked with red bold characters. Bootstrap values (>70%) are shown at the branch nodes. For larger trees, only the lineages or sub-lineages that contain sequences identified in this study are provided.
(TIF)

S1 Table Epidemiological and clinical characteristics of 32 KTR cases. (DOCX)

S2 Table Detailed epidemiological and clinical information on the 32 KTR cases with pneumonia. (XLSX)

S3 Table The clinical signs of the 32 KTR cases with pneumonia. (XLSX)

S4 Table Chest CT results and the descriptions by three clinicians of the 32 KTR cases with pneumonia. (XLSX)

S5 Table Blood cells, lymphocyte subsets, cytokine profiles and biochemical indicators of the 32 KTR cases with pneumonia. (XLSX)

S6 Table Whole blood RNA-seq libraries used in this study and the gene coverage and available read counts. (XLSX)

S7 Table Gene expression matrix (TPM) of the whole blood RNA-seq libraries. (XLSX)

S8 Table Differential expression analysis between control individuals and severe cases. (XLSX)

S9 Table Differential expression analysis between control individuals and critically ill cases. (XLSX)

S10 Table X-Cell enrichment scores of eight immune cells in whole blood RNA-seq libraries. (XLSX)

S11 Table The abundance and diversity of immunoglobulin (Ig) and T cell receptor (TCR) related genes in whole blood RNA-seq libraries. (XLSX)

S12 Table The antimicrobial genes (from GO database) and cytokine genes used in this study. (XLSX)

S13 Table Differential expression analysis for antimicrobial response genes and cytokine genes in whole blood RNA-seq libraries. (XLSX)

S14 Table BALF RNA-seq libraries used for analyzing local immune responses and the gene coverages and available read counts. (XLSX)

S15 Table Gene expression matrix (TPM) of BALF RNA-seq libraries. (XLSX)

S16 Table Differential expression analysis in BALF metatranscriptomes between control individuals and severe cases. (XLSX)

S17 Table Differential expression analysis in BALF metatranscriptomes between control individuals and critically ill cases. (XLSX)

S18 Table X-Cell enrichment scores of eight immune cells and immune score in whole blood RNA-seq libraries. (XLSX)

S19 Table The abundance and diversity of immunoglobulin (Ig) and T cell receptor (TCR) related genes in BALF RNA-seq libraries. (XLSX)

S20 Table Differential expression analysis for antimicrobial response genes and cytokine genes in BALF RNA-seq libraries. (XLSX)

S21 Table Basic information on the DNA-seq and RNA-seq libraries of BALF samples from KTR cases and control individuals. (XLSX)

S22 Table Information on bronchoalveolar lavage fluid (BALF) samples from healthy individuals used in this study. (XLSX)

S23 Table The 56 bacterial genera and their relative abundances in each metagenomic library from BALF samples. (XLSX)

S24 Table The 56 bacterial genera and their relative abundances in each metatranscriptomic library from BALF samples. (XLSX)

S25 Table Shannon and Simpson indices of bacteria at the genus level and present in BALF samples. (XLSX)

S26 Table Read counts of DNA viruses in each DNA-seq library of the BALF samples. (XLSX)

S27 Table Read counts of RNA viruses in each RNA-seq library of the BALF samples. (XLSX)

S28 Table Read counts and relative abundance of fungi in DNA-seq libraries and RNA-seq libraries of BALF samples. (XLSX)

S29 Table Fungal and viral agents (bold red) and their abundance (RPM) in DNA-seq and RNA-seq libraries. (XLSX)

S30 Table Bacterial agents, their pathogenicity and abundance (RPM). (XLSX)

S31 Table Agents identified in the blood samples of these KTR cases and their abundance (RPM). (XLSX)

S32 Table Allelic profiles of SOD, mt26S, CYB, and DHPS genes in *P. jirovecii* identified. (XLSX)

S33 Table Routine clinical microbial detections in 32 KTR cases. (XLSX)

S34 Table Microbial community composition of blank controls in this study (data are shown as relative abundance, %). (XLSX)

S35 Table Bacteria considered as background contamination and their biological characteristics. (XLSX)

S36 Table PCR primers used in this study. (XLSX)

S37 Table Complete or partial genomes of the agents obtained in this study and their genetic features. (XLSX)
