# Supplementary figures and images for: Extensive diversity of unusual microorganisms associated with severe pneumonia in kidney transplant recipients
Source: PLoS Pathog. 2025 Nov 3;21(11):e1013667. doi: 10.1371/journal.ppat.1013667 (PMC12591451; doi:10.1371/journal.ppat.1013667)

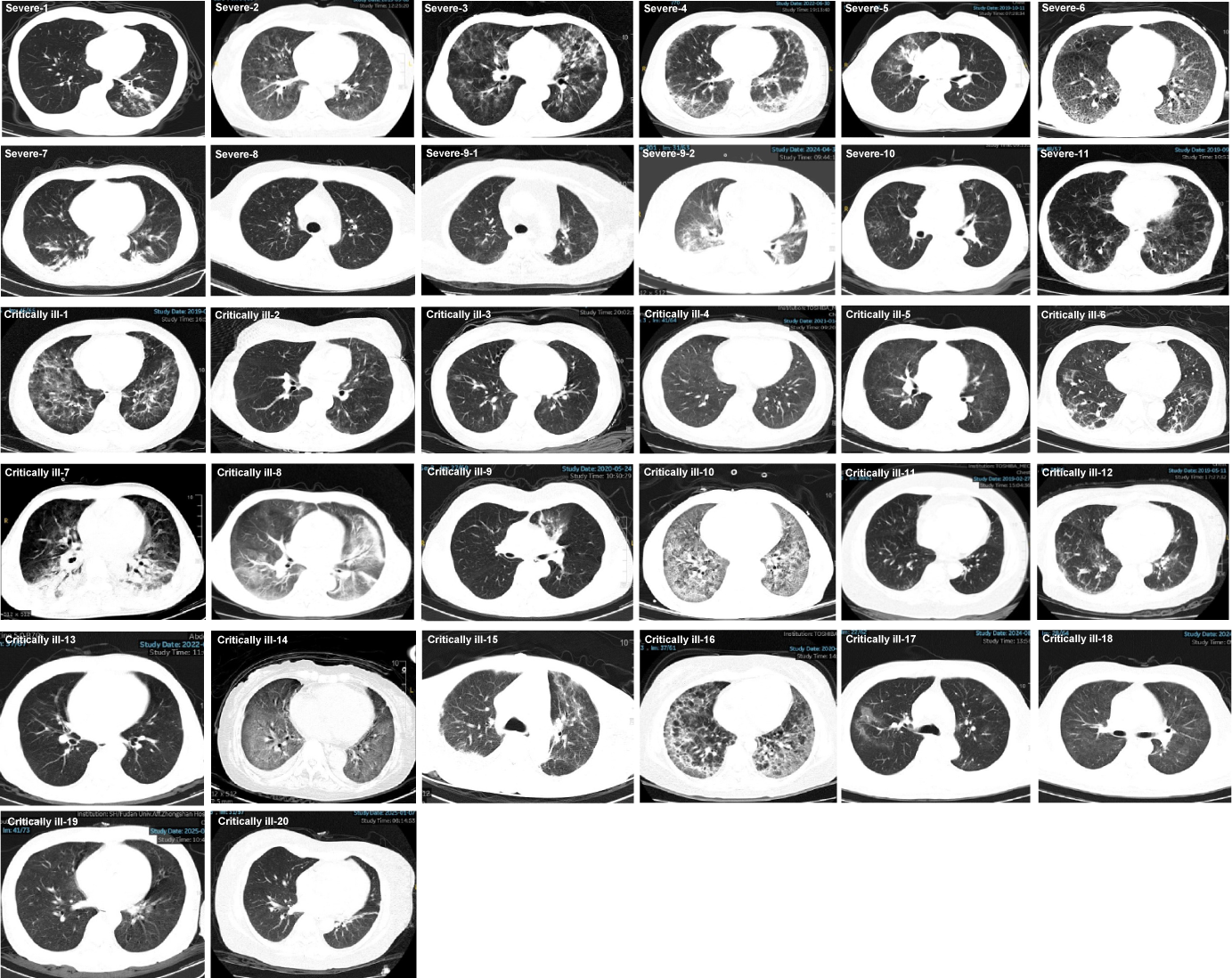

Supplement: S1 Fig — For each case, the most representative CT image on the day of admission is provided. (TIF) [file ppat.1013667.s001.tif]

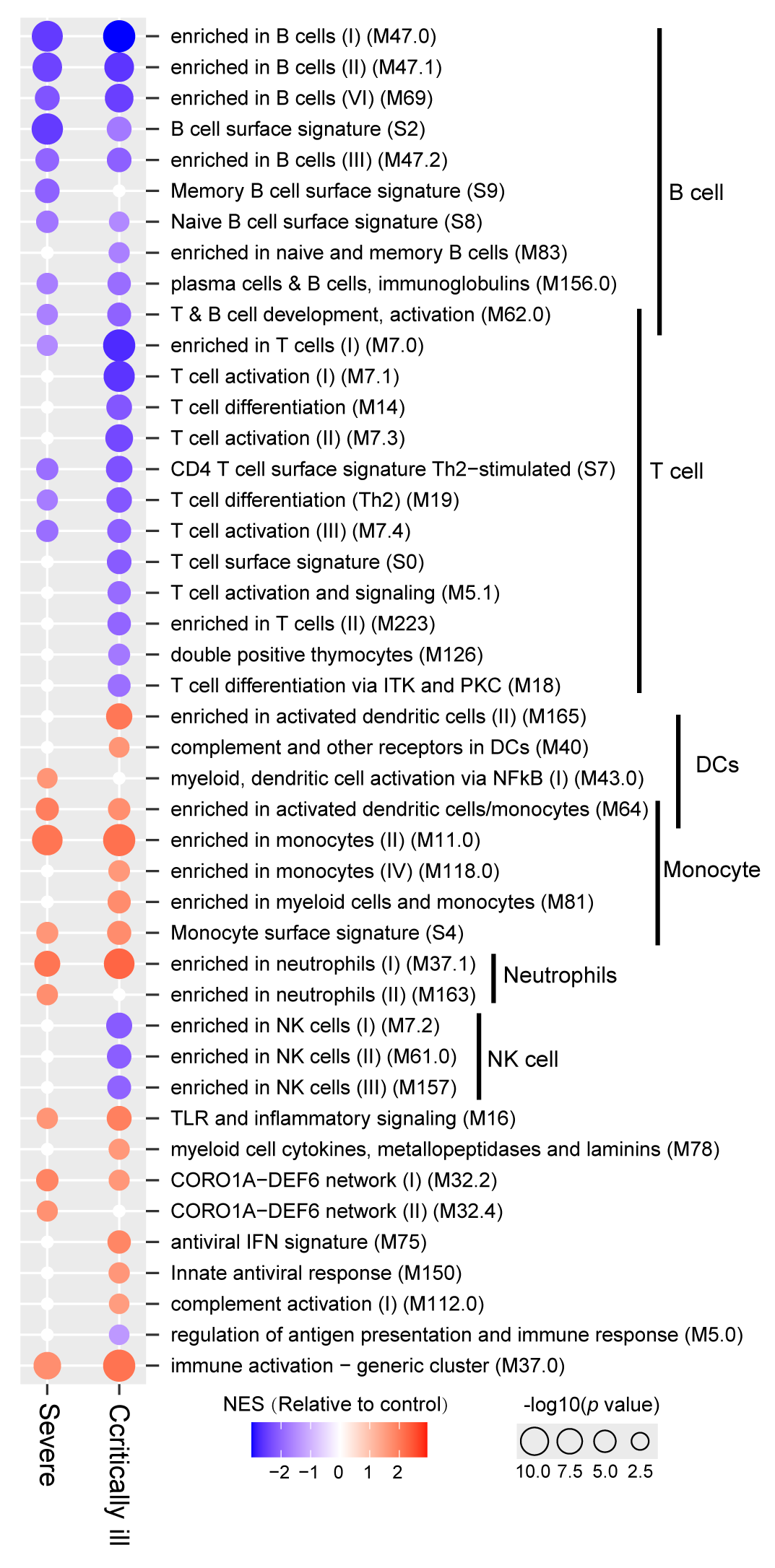

Supplement: S2 Fig — Gene Set Enrichment analyses (FDR < 0.25; 1,000 permutations) were used to identify positive (red), negative (blue), or no enrichment of immune BTMs (gene sets). The color of each point shows the normalized enrichment score (NES) of each BTM selected, and the size of each point shows the p value. (TIF) [file ppat.1013667.s002.tif]

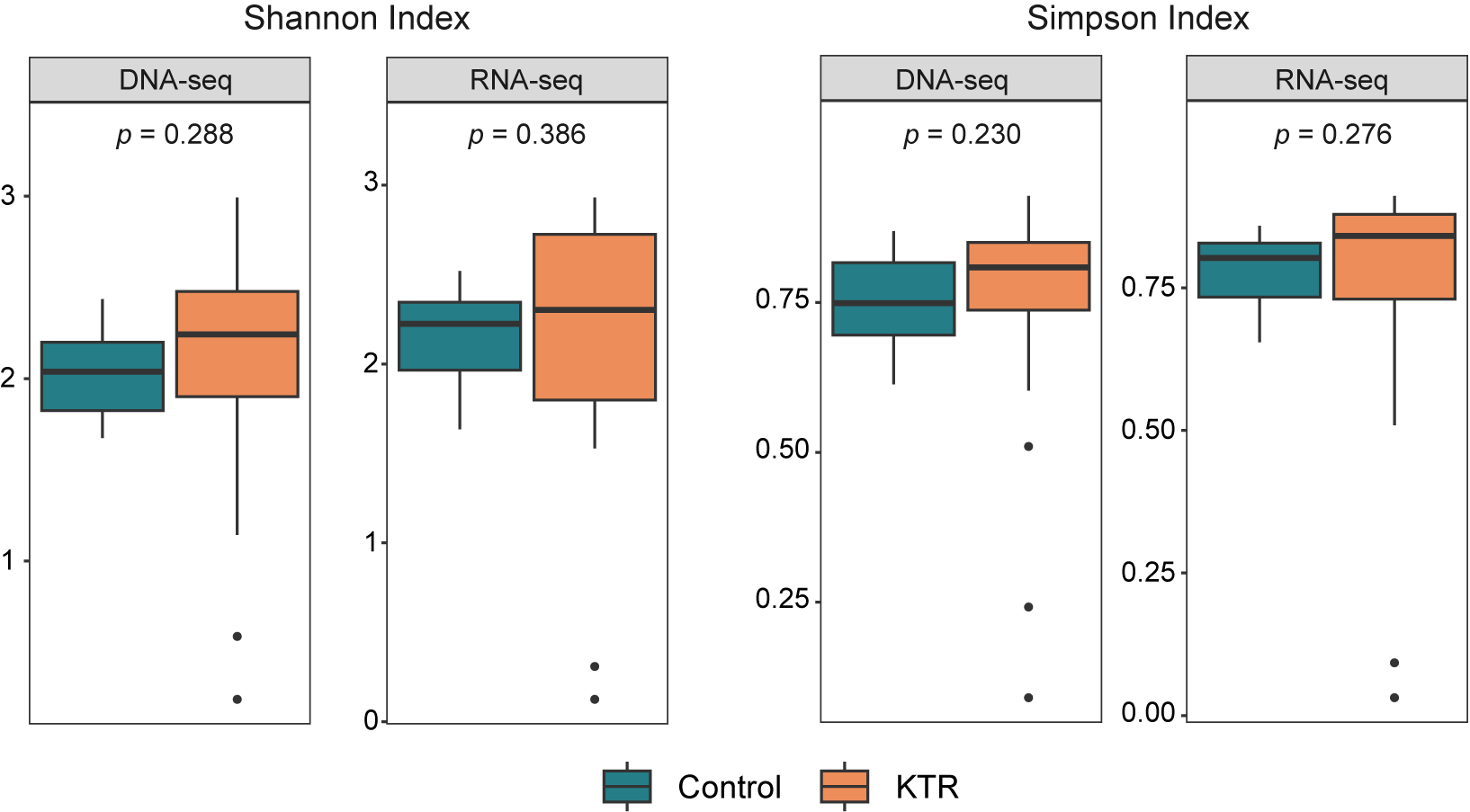

Supplement: S3 Fig — Boxplots showing the Shannon and Simpson indices of the lung bacterial microbiome in KTRs and healthy individuals. These indices were calculated based on BALF metagenome and metatranscriptome data. Differences between groups were tested using a Mann-Whitney U-test. The horizontal box lines in the boxplots represent the first quartile, the median, and the third quartile. The whiskers cover all data points within 1.5 times the inter-quartile range and the black points denote outliers. (TIF) [file ppat.1013667.s003.tif]

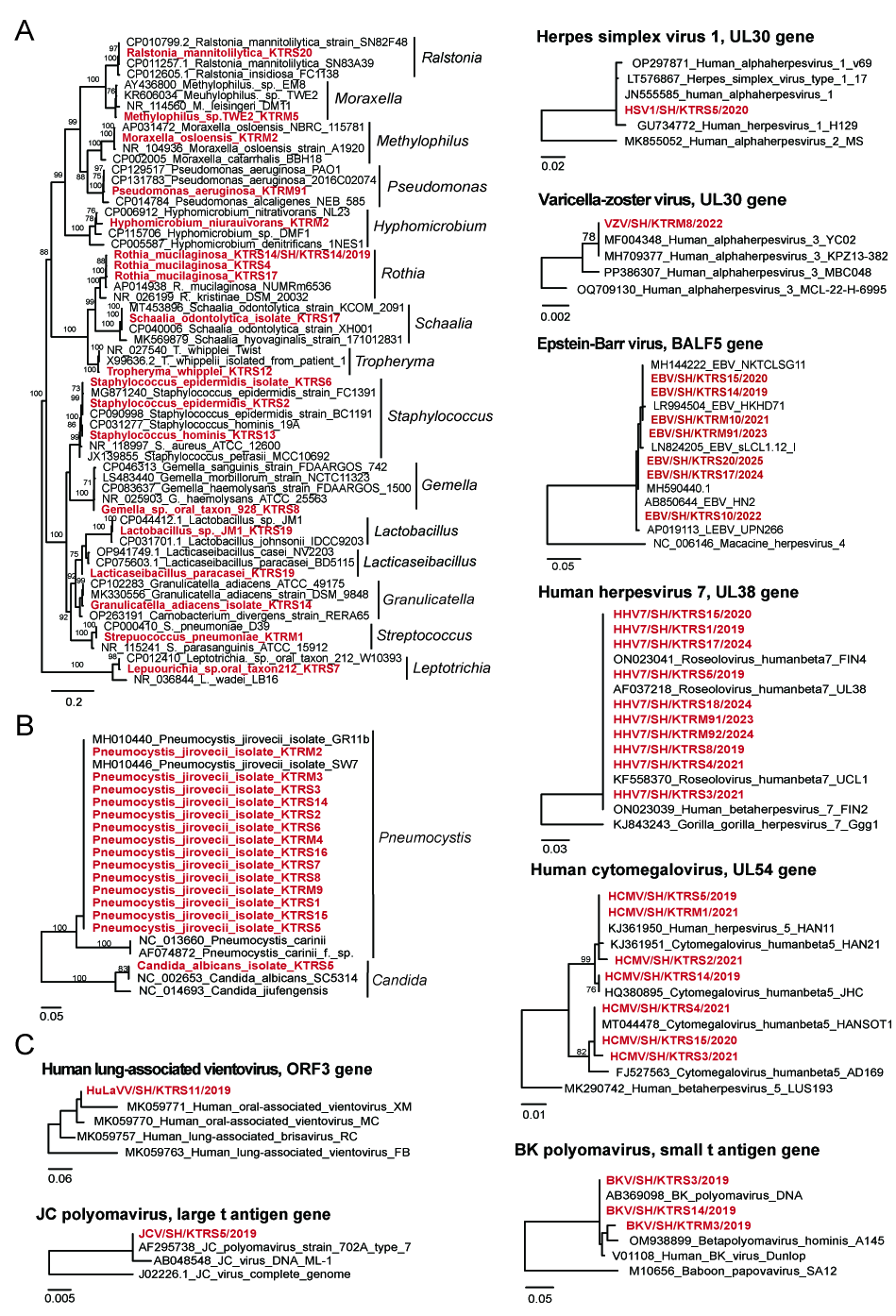

Supplement: S4 Fig — (A) Maximum likelihood phylogenetic tree based on the 16s rRNA gene of the bacterial agents identified here and reference strains from GenBank. (B) Phylogenetic tree based on the cytochrome b gene of the fungi identified here and reference strains from GenBank. (C) Phylogenetic trees based on representative genes of the DNA viruses identified here and reference strains from GenBank. Sequences identified in this study are marked with red bold characters. Bootstrap values (> 70%) are shown at the branch nodes. For larger trees, only the lineages or sub-lineages that contain sequences identified in this study are provided. (TIF) [file ppat.1013667.s004.tif]

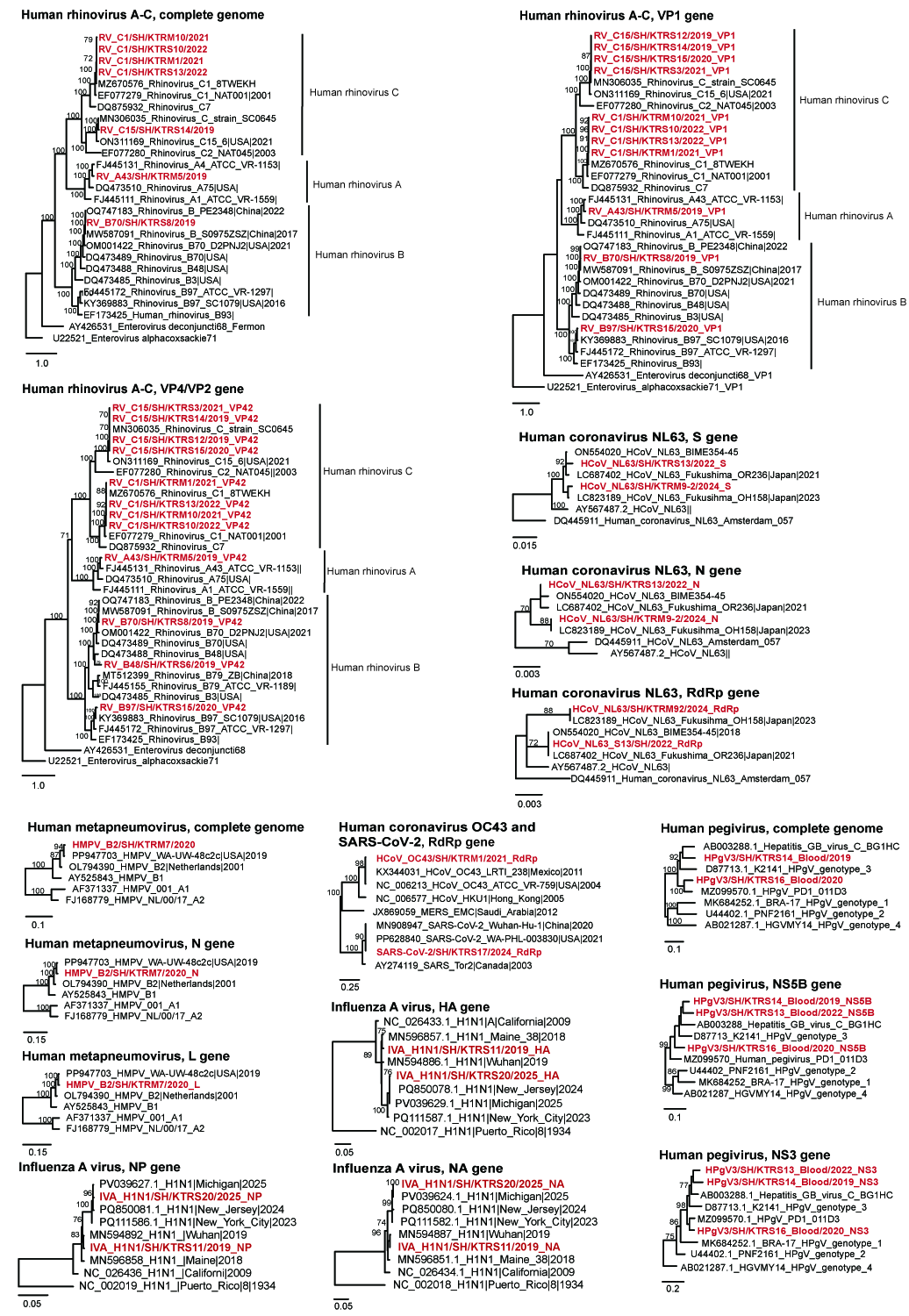

Supplement: S5 Fig — Maximum likelihood phylogenetic trees were estimated based on the complete genome or representative genes of the RNA viruses identified herein. The sequences recovered in this study are marked with red bold characters. Bootstrap values (>70%) are shown at the branch nodes. For larger trees, only the lineages or sub-lineages that contain sequences identified in this study are provided. (TIF) [file ppat.1013667.s005.tif]
