# Supplementary material for: Extensive diversity of unusual microorganisms associated with severe pneumonia in kidney transplant recipients
Source: PLoS Pathog. 2025 Nov 3;21(11):e1013667. doi: 10.1371/journal.ppat.1013667 (PMC12591451; doi:10.1371/journal.ppat.1013667)
Supplement: S1 Table — (DOCX) [file ppat.1013667.s006.docx]

**S1 Table. Epidemiological and clinical characteristics of 32 KTR cases.**

|  | **Total** | **Severe** | **Critically ill** | ***P* value** |
| --- | --- | --- | --- | --- |
| Total number of cases, n | 32 | 12 | 20 |  |
| Female/male | 14/18 | 4/8 | 10/10 | 0.471 |
| Age | 46.50 (36.25-60.00) | 47.50 (33.75-60.75) | 46.50 (36.25-60.00) | 0.924 |
| Hospitalization (day) | 18.50 (13.25-29.75) | 12.50 (10.00-12.50) | 28.50 (17.25-30.75) | 0.255 |
| Maximum temperature (℃) | 38.35 (38.00-39.10) | 38.20 (38.00-38.92) | 38.50 (37.78-39.30) | <0.001 |
| Post-transplantation (month) | 15.50 (7.25-77.00) | 24.00 (8.25-98.75) | 10.50 (6.25-46.75) | 0.950 |
| <6 months, n (%) | 5 (15.63) | 1 (8.33) | 4 (20.00) | 0.626 |
| 6-12 months, n (%) | 9 (28.13) | 3 (25.00) | 6 (30.00) | 1.000 |
| >12 months, n (%) | 18 (56.25) | 8 (66.67) | 10 (50.00) | 0.471 |
| Immunosuppressants, n (%) |  |  |  |  |
| Tacrolimus | 17 (53.13) | 8 (66.67) | 9 (45.00) | 0.291 |
| Cyclosporin A | 9 (28.13) | 1 (8.33) | 8 (40.00) | 0.103 |
| Rapamycin | 6 (18.75) | 3 (25.00) | 3 (15.00) | 0.647 |
| Comorbidities, n (%) |  |  |  |  |
| Hypertension | 22 (68.75) | 7 (58.33) | 15 (75.00) | 0.240 |
| Anemia | 22 (68.75) | 5 (41.67) | 17 (85.00) | 0.006 |
| Diabetes mellitus | 7 (21.88) | 2 (16.67) | 5 (25.00) | 0.683 |
| Coronary heart disease | 2 (6.25) | 0 (0.00) | 2 (10.00) | 0.516 |
| Hepatitis b disease | 3 (9.38) | 2 (16.67) | 1 (5.00) | 0.540 |
| Cancer | 5 (15.63) | 2 (16.67) | 3 (15.00) | 1.000 |
| Clinical signs, n (%) |  |  |  |  |
| Fever | 32 (100.00) | 12 (100.00) | 20 (100.00) | — |
| Cough | 20 (62.50) | 7 (58.33) | 13 (65.00) | 0.724 |
| Expectoration | 8 (25.00) | 2 (16.67) | 6 (30.00) | 0.422 |
| Chest stuff and/or pain | 12 (37.50) | 2 (16.67) | 10 (50.00) | 0.075 |
| Tachypnea and/or dyspnea | 11 (34.38) | 2 (16.67) | 9 (45.00) | 0.139 |
| Hypoxemia | 6 (18.75) | 0 (0.00) | 6 (30.00) | 0.061 |
| Fatigue | 4 (12.50) | 3 (25.00) | 1 (5.00) | 0.136 |
| Anorexia | 3 (9.38) | 2 (16.67) | 1 (5.00) | 0.540 |

Data is shown by n (%) or median (IQR). Significance test was performed using Fisher test. The *p*-value represents statistical significance value between severe and critically ill KTR cases with pneumonia. Bold font denotes statistical significance.
